# Unexpected limited chronic dissection of the ascending aorta

**DOI:** 10.1186/1749-8090-3-49

**Published:** 2008-07-18

**Authors:** Andrea Venturini, Giampaolo Zoffoli, Domenico Mangino, Raimondo Ascione, Alberto Terrini, Angiolino Asta, Gianni Angelini, Elvio Polesel

**Affiliations:** 1 Department of Cardiac Surgery, Ospedale dell'Angelo, Via Paccagnella 11, 30174 Venezia – Mestre, Italy; 2 Bristol Heart Institute, Bristol Royal Infirmary, Marlborough street, Bristol, BS2 8HW, UK

## Abstract

We report a rare case of a limited chronic dissection of the ascending aorta that was accidentally discovered at operation performed for severe aortic stenosis and moderate to severe dilatation of the ascending aorta. Preoperative investigations such as transoesophageal echocardiography and cardiac catheterization missed the diagnosis of dissection. Intraoperative findings included a 3.5 cm eccentric bulge of the ascending aorta and a 5 mm circular shaped intimal tear comunicating with a limited hematoma or small dissection of the media layer. (The rarety of the report is that the chronic dissection is limited to a small area (approximatively 3.5 × 2.5 cm) of the ascending aorta).

## Introduction

Aortic dissection is defined as the separation of the lamellae of the aortic wall. The extent of undermining of the intimal layer by dissection can vary from being only a few millimetres to extending to all the aorta itself. The dissecting process creates a false lumen that may vary from only a few millimetres to the larger classic false lumen [1]. Chronic dissections of the ascending aorta are very rare because of the catastrophic natural history of the acute ones. Among chronic dissections of the ascending aorta, report of a very limited extension of the haematoma is only anecdotal [2].

## Case report

A 83 years-old woman was referred to our department for surgical treatment after being diagnosed a severe aortic stenosis associated with a dilatation of the ascending aorta.

The patient had recently been hospitalized for congestive heart failure. Patient history included permanent atrial fibrillation and no previous episodes of chest or back pain suggestive of aortic dissection.

**Figure 1:**
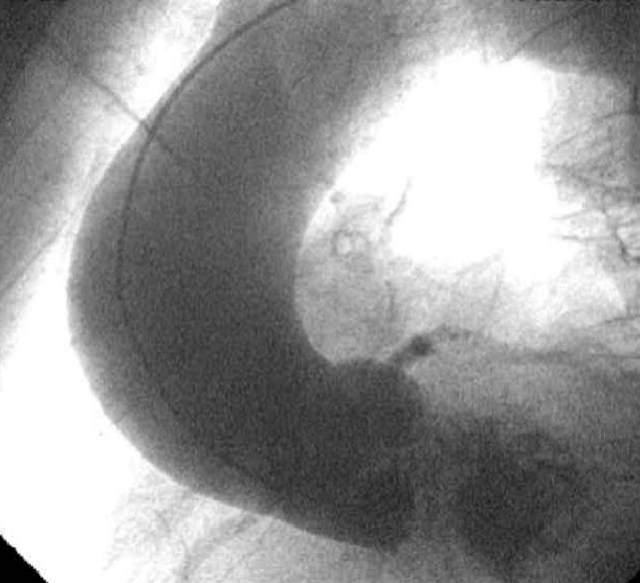
Preoperative aortography showing the dilatation of the ascending aorta without obvious evidence of limited dissection.

Chest X ray showed a slightly enlarged mediastinum and moderate calcifications of the aortic arch; a transthoracic echocardiogram revealed a severe aortic stenosis (aortic valve area: 0.6 cm^2^) together with moderate aortic and tricuspid valve regurgitation. Cardiac catheterization showed a markedly calcified aortic valve with moderate to severe insufficiency and a 4.5 to 5 cm dilatation of the ascending aorta above the sino-tubular junction without any other pathologic findings including the presence of an eccentric bulge; coronary arteries were normal (Figure. 1).

**Figure 2:**
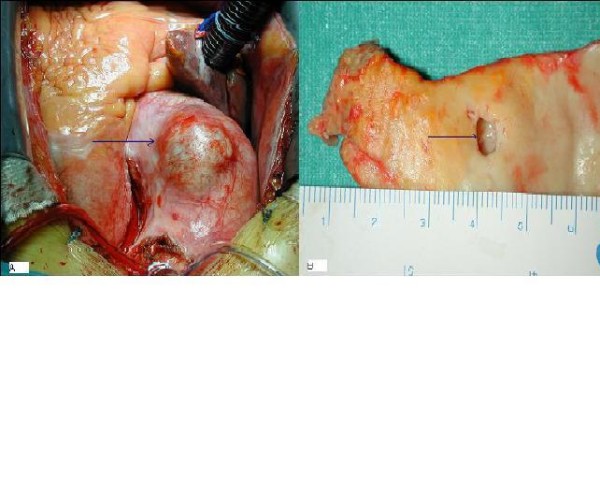
a) **At opening of the pericardium is present an eccentric bulge of the ascending aorta with a very thin adventitial layer**. b) Aortic wall showing the 5 mm chronic intimal tear leading into the limited dissection.

Operation was performed one month after the cardiac catheterization. Intraoperative transoesophageal echocardiography confirmed the findings of the trans-thoracic examination. A marked biatrial dilatation was also observed and therefore the possibility to perform a radiofrequency ablation of the atrial fibrillation was not taken into consideration. Intraoperative examination showed a 3.5 cm eccentric bulge of the anterior portion of the ascending aorta (Figure. 2-a).

Cardiopulmonary bypass was instituted and cardioplegic arrest with selective antegrade Custodiol^® ^(Dr. F. Koehler Chemie Gmbh, Alsbach-Haenlein) cardioplegia was obtained. The aneurysm of the ascending aorta was resected and inspected: there was a 5 mm circular shaped intimal tear comunicating with a limited hematoma or small dissection of the media layer (Figure. 2-b). The aortic valve was tricuspid with massive calcifications; the aortic root was not dissected or dilated. After explantig the aortic valve a 21 mm Hancock II^® ^(Medtronic Inc., Minneapolis, Minnesota) porcine bioprostheses was inserted. The ascending aorta was then replaced with a 30 mm dacron tube graft, the proximal anastomosis being at the level of the sino-tubular junction and the distal anastomosis being just below the innominate artery. The patient was weaned off the cardiopulmonary bypass easily and made an uneventful recovery. Aortic cross clamp and cardiopulmonary bypass time were respectively 85 and 91 minutes.

Transthoracic echocardiograms and general examination performed three and nine months after operation were unremarkable.

Hystologic examination of the aortic wall showed dissection associated with thrombosis of the media layer while chronic inflammation and fibrosis of the adventitia were detected.

## Discussion

According to the classification proposed by L.G. Svensson in 1999 there are five variants of aortic dissection: classic type (class 1: association of intimal tear and presence of dual lumens); intramural hematoma (class 2); intimal tear without hematoma (limited dissection) and eccentric aortic bulge (class 3); atherosclerotic penetrating ulcers (class 4); iatrogenic/traumatic dissection (class 5) [2].

We want to focus our attention to class 3 variant in which the intimal tear is associated with exposure of the aortic media or adventitial layer but without extensive separation of medial layers.

Because of this limited pathology the current imaging techniques may be inadequate for the diagnosis of this type of dissection. Svensson, in his article regarding intimal tear without hematoma, reported that all the 9 patients with limited dissection in a series of 181 consecutive patients were not diagnosed preoperatively. A careful review of preoperative investigations revealed the presence of an eccentric aortic bulge on the aortography in 5 of the nine patients while this bulge was not noted on the transoesophageal studies [2]. In patients with limited dissection the very thin outer adventitial layer may rupture resulting in cardiac tamponade as happened in 3 of the 9 patients of the previously mentioned group.

This type of dissection may propagate to become the classic type 1 dissection as described by Svensson and Gott. These authors reported older healed class 3 intimal tears associated with acute dissections, particularly in Marfan patients [3,4].

Our report is consistent with the up to date literature because our limited dissection was not diagnosed preoperatively, too. Therefore we speculate that if the patient was not taken to theatre because of the severe aortic stenosis, then probably the very thin adventitia would have ruptured or an acute dissection would have occurred. As a matter of fact we know that at least one third of the patients with aortic dissection are not diagnosed before death [5].

In conclusion our small limited dissection eluded two of the most current imaging modalities yet have catastrophic consequences if unrecognized. Newer tridimensional imaging techniques could be helpful in the diagnosis and treatment of aortic aneurysms.

## Authors' contributions

Each author contributed equally to the paper. All authors read and approved the final manuscript.

## Consent

Written informed consent was obtained from the patient for publication of this Case report and any accompanying images. A copy of the written consent is available for review by the Editor-in-Chief of this journal.
